# On the Application of Homotopy Perturbation Method for Solving Systems of Linear Equations

**DOI:** 10.1155/2014/143512

**Published:** 2014-11-16

**Authors:** S. A. Edalatpanah, M. M. Rashidi

**Affiliations:** ^1^Department of Applied Mathematics, Islamic Azad University, Tonekabon Branch, Tonekabon, Iran; ^2^Mechanical Engineering Department, Engineering Faculty of Bu-Ali Sina University, Hamedan, Iran

## Abstract

The application of homotopy perturbation method (HPM) for solving systems of linear equations is further discussed and focused on a method for choosing an auxiliary matrix to improve the rate of convergence. Moreover, solving of convection-diffusion equations has been developed by HPM and the convergence properties of the proposed method have been analyzed in detail; the obtained results are compared with some other methods in the frame of HPM. Numerical experiment shows a good improvement on the convergence rate and the efficiency of this method.

## 1. Introduction

Consider the following convection-diffusion equation:

(1) $$\begin{array}{c} -\underset{\Delta u}{\underset{︸}{\left(u_{xx}+u_{yy}\right)}}+\delta u_{x}+\tau u_{y}=f\left(x,y\right). \end{array}$$

On the unit square domain *Ω* = [0,1] × [0,1], with constant coefficients *δ*, *τ*, subject to Dirichlet boundary conditions. Discretization by a five-point finite difference operator leads to the following linear system:

(2) $$\begin{array}{c} AX=b, \end{array}$$

where *X* denotes a vector in a finite-dimensional space and *A* ∈ *R* ^*n*^2^×*n*^2^^. With discretization on a uniform *n* × *n* grid, using standard second-order differences for the Laplacian and either centered or upwind differences for the first derivatives, the coefficient matrix has the form

(3) A=tridiagonal[bI,tridiagonal[c,a,d],eI],mmmcb=−1+∂; c=−1+γ; a=4;mmmcmmcd=−1−γ; e=−1−∂.

Or with Kronecker product (⊗) we obtain

(4) A=I⊗Tx+I⊗Ty,Tx=tridiagonal[c,a,d],Ty=tridiagonalbI,0,eI,

where ∂ = *τh*/2; *γ* = *δh*/2 are Reynolds numbers. Also, the equidistant step-size *h* = 1/*n* is used in the discretization and the natural lexicographic ordering is employed to the unknowns and the right hand side satisfies *b*_*ij*_ = *h* ^2^ *f*_*ij*_(*x*, *y*). For details, we refer to [1, 2] and the references therein. Convection-diffusion equations appear in many fields of science and engineering and there are some reliable methods for solving this class of problems ([1, 2] and the references therein). Here, we use alternative approach to solve (2) based on homotopy perturbation method (HPM). The HPM was first proposed by the Chinese mathematician He in 1999 [3] and was further developed and improved by him [4–6]. He presented a homotopy perturbation technique based on the introduction of homotopy in topology coupled with the traditional perturbation method for the solution of algebraic equations and ODEs. This technique provides a summation of an infinite series with easily computable terms, which converges to the solution of the problem. In the literature, various authors have successfully applied this method for many kinds of different problems such as nonlinear partial differential equations [7, 8], nonlinear wave equations [5], nonlinear integral and integrodifferential equations [9], fractional IVPs [10], and optimization [11]. Nevertheless, to our knowledge, a few papers have considered this analytical method for system of linear equations. Keramati [12] presented an efficient method for solving system of linear equations based on HPM. Liu [13] by using HPM and splitting methods proposed a new method for linear systems. Saberi Najafi et al. [14] based on HPM proposed a new algorithm for fuzzy linear systems and compared it with Adomian's decomposition method. They also in [15] show that solving linear systems by using a new method called modified HPM, presented by Noor et al. [16], is impractical.

In this paper, by combination of HPM and preconditioning technique, we will present a new model for solving linear systems and apply this method to the above convection-diffusion equation.

## 2. Homotopy Perturbation Method for Linear Systems

Consider linear system (2). HPM procedure for solving of this system is a continuous map from the interval [0,1] to a function space where it is as follows:

(5) $$\begin{array}{c} H\left(u,p\right)=\left(1-p\right)F\left(u\right)+pL\left(u\right)=0, \end{array}$$

and *p* ∈ [0,1] is an embedding parameter.

Keramati [12] applied a HPM to solve linear systems by the following definitions.

Let *L*(*u*) = *Au* − *b* and *F*(*u*) = *u* − *w*_0_, where *w*_0_ is a known vector. Then homotopy *H*(*u*, *p*) is

(6) $$\begin{array}{c} H\left(u,p\right)=\left(1-p\right)\left(u-w_{0}\right)+p\left(Au-b\right)=0. \end{array}$$

Obviously, we will have

(7) $$\begin{array}{c} H\left(u,0\right)=F\left(u\right),\;\;H\left(u,1\right)=L\left(u\right). \end{array}$$

According to the HPM, we can first use the embedding parameter *p* as a small parameter and assume that the solution of (2) can be written as a power series in *p*:

(8) $$\begin{array}{c} u=u_{0}+pu_{1}+p^{2}u_{2}+\cdots , \end{array}$$

and the exact solution is obtained as follows:

(9) $$\begin{array}{c} x=\underset{p\to 1}{\lim}u=\underset{p\to 1}{\lim}\left(u_{0}+pu_{1}+p^{2}u_{2}+p^{3}u_{3}+\cdots \right)=\overset{\infty }{\underset{j=0}{\sum }}u_{j}. \end{array}$$

Putting (8) into (5) and comparing the coefficients of identical degrees of *p* on both sides, we find

(10) p0:u0=w0p1:A−Iu0+u1−w0−b=0,u1=b−A−Iu0+w0,p2:A−Iu1+u2=0,u2=−A−Iu1,⋮.

And in general

(11) $$\begin{array}{c} u_{n+1}=-\left(A-I\right)u_{n},\;n=1,2,\ldots . \end{array}$$

Taking *u*_0_ = *w*_0_ = 0 yields

(12) u1=b,u2=−(A−I)u1=−(A−I)b,u3=−(A−I)u2=A−I2b,⋮un+1=−(A−I)un=−1nA−Inb.

Therefore, the solution can be of the form

(13) $$\begin{array}{c} x=\overset{\infty }{\underset{i=0}{\sum }}{\left(-1\right)}^{i}{\left(A-I\right)}^{i}b. \end{array}$$

This method is efficient for solving system of linear equations but, however, does not converge for some systems when the spectral radius is greater than one. To make the method useful, Liu in [13] added the auxiliary parameter and the auxiliary matrix to the HPM. This method is as follows:

(14) $$\begin{array}{c} H\left(u,p\right)=\left(1-p\right)F\left(u\right)+\left(qH\right)pL\left(u\right)=0, \end{array}$$

where

(15) $$\begin{array}{c} L\left(u\right)=Au-b,\;\;F\left(u\right)=Qu-w_{0}. \end{array}$$

Here, *q* is an auxiliary parameter, *H* is the auxiliary matrix, and the operator *F*(*u*) is decided by the splitting matrix *Q*. Then his iterative scheme for *u* is

(16) $$\begin{array}{c} u_{n+1}={\left(I-qQ^{-1}HA\right)}^{n}\left(qQ^{-1}H\right)b. \end{array}$$

Therefore,

(17) $$\begin{array}{c} u=\overset{\infty }{\underset{i=0}{\sum }}u_{i}p^{i}=\overset{\infty }{\underset{i=0}{\sum }}\left[{\left(I-qQ^{-1}HA\right)}^{i}\left(qQ^{-1}H\right)b\right]p^{i}. \end{array}$$

And finally by setting *p* = 1, he obtained the solution as follows:

(18) $$\begin{array}{c} u=\overset{\infty }{\underset{i=0}{\sum }}u_{i}=\overset{\infty }{\underset{i=0}{\sum }}{\left(I-qQ^{-1}HA\right)}^{i}\left(qQ^{-1}Hb\right). \end{array}$$

This author has adapted the Richardson, Jacobi, and the Gauss-Seidel methods to choose the splitting matrix and obtained that the homotopy series converged rapidly for a large sparse system with a small spectral radius. But to improve the rate of convergence, he does not present on a method for choosing *H* (see [13], conclusion). The main goal on this paper is studying this problem and doing some modifications for having very effective and straightforward results.

Our idea for choosing *H* is preconditioning technique. Preconditioning methods are the most authoritative techniques to improve poor properties of the basic iterative methods. The main aim of preconditioning method for *AX* = *b* is to substitute the original matrix *A* with an equivalent one *A* ^prec^ which has better properties concerning the computation of a solution (generally by a certain iterative method). The two matrices are equivalent in the sense that they have the same solution. Simple preconditioners of this type are the left matrix preconditioners. The left preconditioned matrix is a nonsingular matrix *M* and the preconditioned matrix is defined by *A* ^prec^ = *MA*, where *M* ≈ *A* ^−1^.

In this paper, we consider *H* as a preconditioner and if the auxiliary matrix *H* is properly chosen, we can be able to achieve the best results. To improve the convergence rate of the basic iterative method, various models of preconditioning systems have been proposed [17–19]. In the literature, various authors have suggested different models of (*I* + *S*)-Type preconditioned [20–28], where all of these models are left preconditioners. We can consider all of the models in [17–28] for *H*.

Here we consider Usui et al. [22] model of (*I* + *S*)-Type preconditioners for *H*, where

(19) $$\begin{array}{c} H={\left(h_{ij}\right)}_{n\times n}=\left\{\begin{array}{cc} 1, & j=i,\\ -\frac{a_{ij}}{a_{jj}}, & j>i,\\ 0, & \text{otherwise}. \end{array}\right. \end{array}$$

Let *A* = *D* − *L* − *U*, where *D* is the diagonal matrix and *L*, *U* are strictly lower and strictly upper triangular matrices of *A*, respectively. Then the preconditioned matrix is as follows:

(20) Aprec=HLu=HAu−bprec,bprec=Hb.

We know that *H* = (*I* + *S*), where

(21) $$\begin{array}{c} S=\left(s_{ij}\right)=\left(-\frac{a_{ij}}{a_{jj}}\right),\;\forall j>i. \end{array}$$

Then we have

(22) HA=(I+S)(D−L−U)=(D−L−U)+SD−SL−SU.

Thus,

(23) $$\begin{array}{c} HA=\left(D-D_{1}\right)-\left(L+L_{1}\right)-\left(U-SD+U_{1}+SU\right), \end{array}$$

where *D*_1_, *L*_1_, and *U*_1_ are, respectively, the diagonal, strictly lower, and strictly upper triangular parts of *SL* = *D*_1_ + *L*_1_ + *U*_1_.

Therefore, we have

(24) D−=D−D1,L−=L+L1,U−=U−SD+U1+SU.

Furthermore, for more convergence speed in Jacobi-HPM and Gauss-Seidel-HPM we choose *Q* as follows.

In Jacobi-HPM,

(25) $$\begin{array}{c} Q=\overset{-}{D}. \end{array}$$

In Gauss-Seidel-HPM,

(26) $$\begin{array}{c} Q=\overset{-}{D}-\overset{-}{L}. \end{array}$$

Now, we will show that by this choice of *H*, *Q* these methods are faster than of the basic methods form point of view of the convergence speed.

**Definition 1 (see [29, 30]).**

(a) A matrix *A* = *a*_*ij*_ is called a *Z*-matrix if for any *i* ≠ *j*, *a*_*ij*_ ≤ 0.

(b) A *Z*-matrix is an *M*-matrix, if *A* is nonsingular and *A* ^−1^ ≥ 0.

**Definition 2 (see [29, 30]).**

Let *A* be a real matrix. The splitting *A* = *M* − *N* is called

(a) convergent if *ρ*(*M* ^−1^ *N*) < 1 (*ρ*(·) is the spectral radius of matrix);
(b) regular if *M* ^−1^ ≥ 0 and *N* ≥ 0.

**Lemma 3 (see [29]).**

Let A be a *Z*-matrix. Then *A* is *M*-matrix if and only if there is a positive vector *X* such that *AX* > 0.

**Lemma 4 (see [31]).**

Let *A*, *B* be *Z*-matrix and *A* an *M*-matrix; if *A* ≤ *B*, then *B* is an *M*-matrix too.

**Lemma 5 (see [32]).**

Let *A* = *M* − *N* be a regular splitting. Then *ρ*(*M* ^−1^ *N*) < 1 if and only if *A* is nonsingular and *A* ^−1^ is nonnegative.

**Lemma 6 (see [24], Theorem 2.7, Remark 2.20).**

Let *A* = [*D* − (*L* + *U*)] = [(*D* − *L*) − *U*] and $HA=[\overset{-}{D}-(\overset{-}{L}+\overset{-}{U})=(\overset{-}{D}-\overset{-}{L})-\overset{-}{U}]$ be regular splittings of *A* and *HA* where *H* is (19). If *A* is *M*-matrix, then

(27) $$\begin{array}{c} \rho \left({\overset{-}{D}}^{-1}\left(\overset{-}{L}+\overset{-}{U}\right)\right)\leq \rho \left(D^{-1}\left(L+U\right)\right)<1, \end{array}$$

(28) $$\begin{array}{c} \rho \left({\left(\overset{-}{D}-\overset{-}{L}\right)}^{-1}\overset{-}{U}\right)\leq \rho \left({\left(D-L\right)}^{-1}U\right)<1. \end{array}$$

By applying Lemma 3, we can achieve the following results.

**Theorem 7 .**

Let *A* be *M*-matrix. If we use *H* in (19) and *Q* in (25) and (26), then convergence rate of (18) is faster than of the convergence rate in $\sum_{i=0}^{\infty }{\left(I-q{\hat{Q}}^{-1}A\right)}^{i}(qQ^{-1}b)$, $(\hat{Q}=D$ or $\hat{Q}=D-L)$.

**Proof**

Since *A* is an *M*-matrix, by Lemma 3 it is easy to see that *HA* also is *M*-matrix, and thus by Lemma 4 *Q* is *M*-matrix and *Q* − *qHA* is nonnegative. Then by Lemmas 5 and 6 we can obtain $\rho (I-qQ^{-1}HA)\leq \rho (I-q{\hat{Q}}^{-1}A)<1$.

Therefore by [13, Theorem 2.1] the proof is completed.

## 3. Numerical Experiments

In this section, we give an example to illustrate the obtained results in the previous sections.

We test convection-diffusion equation (1) when *δ* = 1, *τ* = 2.

Then, we solved the *n* ^2^ × *n* ^2^ *M*-matrix yielded by Jacobi-HPM and Gauss-Seidel-HPM.

In this experiment, we choose the right hand side vector, such that *X* = (1,1,…,1)^*T*^ is solution of *AX* = *b*. The stopping criterion with tolerance *ε* = 10^−10^ is ‖*Au* − *b*‖ < *ε*.

In Tables 1 and 2 we report the* CPU* time and number of iterations (*Iter*) for the Jacobi-HPM and Gauss-Seidel-HPM methods, respectively.

From the tables, we can see that if *H* is (19), then the mentioned methods are superior to the basic methods.

## 4. Conclusions

In this paper, we have applied a new method for solving convection-diffusion equations based on the HPM. We can see that how HPM method is influenced for solving system of linear equation, if combined by preconditioning technique. Finally, from theoretical speaking and numerical experiment, it may be concluded that the convergence behaviors of this method is superior to some other methods in the frame of HPM.

## Figures and Tables

**Table 1:** The results of experiment for Jacobi-HPM.

Method		Algorithm for *q* = 1, *H* = *I*		Algorithm for *q* = 1, *H* = (*I* + *S*) in (19)	
*n*	*N*	Iter	CPU	Iter	CPU
5	25	**42**	0.015893	**24**	0.003804
10	100	**63**	0.501852	**37**	0.231103
15	225	**81**	8.018167	**47**	3.844972
20	400	**96**	71.971452	**56**	35.328229

**Table 2:** The results of experiment for Gauss-Seidel-HPM.

Method		Algorithm for *q* = 1, *H* = *I*		Algorithm for *q* = 1, *H* = (*I* + *S*) in (19)	
*n*	*N*	Iter	CPU	Iter	CPU
5	25	**24**	0.007968	**10**	0.001203
10	100	**38**	0.255449	**17**	0.069256
15	225	**51**	4.428433	**22**	1.424873
20	400	**62**	40.541738	**27**	13.336331
